# Repellent Effect of the Caraway *Carum carvi* L. on the Rice Weevil *Sitophilus oryzae* L. (Coleoptera, Dryophthoridae)

**DOI:** 10.3390/insects11120836

**Published:** 2020-11-26

**Authors:** Małgorzata Kłyś, Aleksandra Izdebska, Natalia Malejky-Kłusek

**Affiliations:** Department of Ecology and Environmental Protection, Institute of Biology, Pedagogical University of Cracow, Podchorążych 2, 30-084 Kraków, Poland; natalia.malejky@o2.pl

**Keywords:** essential oil, insecticides, L-carvone, emigration, storage pests

## Abstract

**Simple Summary:**

*Sitophilus oryzae* is one of the main insects that attack stored products around the world. Effective and environmentally friendly substances for managing storage pests are still being sought. Natural plant substances are tested as an alternative to chemical methods. Therefore, laboratory tests were carried out to check the effect of various concentrations of caraway essential oil and L-carvone on rice weevil. The abundance, emigration and mortality of this species were recorded. The highest repellent effect and the highest mortality of *Sitophilus oryzae* were caused by 0.1% L-carvone and 0.5% caraway essential oil. Lower concentrations of the tested compounds showed stronger repellant properties than higher concentrations.

**Abstract:**

The aim of the study was to check the effect of *Carum carvi* L. essential oil and L-carvone on the emigration, repellence and mortality of *Sitophilus oryzae* L. In the experiments with caraway essential oil, concentrations of 0.1%, 0.5% and 1% were used, and concentrations of 0.05, 0.1, 0.5 and 1% were used with L-carvone. We assessed whether, in what concentrations and after what exposure time the substances acted on *S. oryzae* as repellents and/or insecticides. The laboratory tests were carried out at 29 ± 1 °C with 60 ± 5% relative humidity (RH). The deterrence, mortality and abundance of insects were noted after 1, 2, 3, 4, 5, 24 and 48 h. For caraway essential oil and L-carvone, the highest repellency was not shown in the highest concentrations that were used in the tests but in the lower concentrations of 0.5% and 0.1%, respectively. In all used concentrations, caraway essential oil showed repellent effects on *S. oryzae*. The highest repellency (60–98%) was caused by 0.5% caraway essential oil after 1, 2, 3, 4 and 5 h of research and by 0.1% L-carvone (16–100%). The highest mortality of *S. oryzae* was caused by 0.5% caraway essential oil and 1% L-carvone. L-carvone at a concentration of 0.05% did not cause mortality in *S. oryzae.* In conclusion, the greatest repellent effects on *S. oryzae* were caused by lower doses of caraway essential oil and L-carvone. These compounds do not show the normal relationship described in the previous literature on warehouse pests, in which the repellency increased with increasing concentrations of the tested plant material.

## 1. Introduction

It is expected that by 2050 the number of people in the world will increase to 9.1 billion and that to feed that number an additional 70% increase in food production will be needed [1,2,3]. Such an increase in human population requires that one secure its food demands, and this poses a major challenge for all of humanity. These concerns are further enhanced by the development of urbanisation, taking land for nonagricultural uses, and by climate changes. An increased food production can be achieved not only by increases in cultivated areas, but also through activities involving cultivation, the protection of crops against agrophages and the proper harvesting of crops followed by appropriate storage [4].

The losses in world food production are enormous. At each stage of production, from producers to consumers, there are losses that constitute enormous “chains of losses.” It is estimated that 30–50% (1.2–2 billion tonnes) of produced food is lost each year [4]. Gitonga et al. [5] and Lesk et al. [6] report that biotic factors (insects, mites, rodents and fungi) and abiotic factors (temperature, humidity) cause losses in the total harvest even reaching up to 60%. A large number of publications indicate that the greatest losses are incurred during storage, particularly in developing countries [7,8,9,10]. In developed countries, the over-production of food is frequent, and storing the surpluses creates favourable conditions for the development of pests. The damage during storage can either be direct (loss in the mass of products) or indirect (reduction in terms of a lower quality and nutritive value), in addition to a reduced grain germination capacity. Every year, 5–10% of produced food is lost during storage only [4].

Over 20 thousand species of field and storage pests are responsible for destroying approx. one-third of the world food production [11]. Most of these species spread everywhere where food and favourable conditions for development can be found. One such dangerous pest of stored cereal grain, e.g., wheat, maize, and rice, is *Sitophilus oryzae* [12]. Srivastava et al. [13] also list *S. oryzae* alongside *Rhyzopertha dominica* as a dangerous and one of the most frequent pests of stored rice grain. These insects bring about a reduction in germination capacity, reduction in nutritive value and changes in the chemical composition of grains, etc.

A decrease in great losses occurring after harvests can lead to an increased availability of food (which reduces world hunger) and a decreased pressure exerted on natural resources, particularly renewable resources. Currently, Integrated Pest Management (IPM) is the group of methods that is being increasingly selected for pest control because they are safe, environmentally friendly and economic ways of controlling storage pests [14]. The application of synthetic chemical insecticides raises a number of doubts associated with their adverse effects upon the environment and human health [11,15,16,17]. Using natural products is both effective and beneficial to the environment. Such substances include powders, oils and plant extracts [15,18,19].

Recently, there has been an intensification of laboratory research on essential oils (EOs) and their ingredients in order to use their properties to control many pests of agricultural crops and horticulture, as well as pests of storage products. In many studies it was found that EOs and compounds isolated from them reduced egg laying and adult-stored pests’ emergence, increased their mortality, deterred pests from products and had repellent properties [16,19,20,21,22,23]. In addition, biopesticides of plant origin, including EOs, have favorable, desirable properties. For instance, EO is composed of many different compounds whose mechanism of action prevents the evolution of pest resistance. Additionally, EOs are rarely toxic to mammals. They have a short shelf life and are therefore considered environmentally safe [24,25,26].

Furthermore, the efficacy of EO can be increased by using it in synergistic combinations with other ecofriendly agents. It is already known that the combination of different compounds from EOs may have an additive, synergistic or antagonistic effect on a particular pest [26,27]. Mixtures of compounds increase the insecticidal spectrum of action as different species have different responses to individual compounds [26]. The synergy of action of plant pesticides used in agriculture gives promising results. A stronger deterrent effect is shown by lower concentrations of mixed compounds of plant pesticides, delaying the development of resistance to these substances among pest species [28].

It should be noted that plant-based products (including EOs) also have weaknesses, as well as some limitations in terms of their large-scale use and their commercialization. For instance, they a show poor stability (short environmental persistence) and lower efficacy than chemical insecticides, and they pose other technological problems. In addition, their toxicological and environmental assessments are expensive, and they must also comply with strict registration regulations [29,30,31]. Therefore, relatively few of them have been registered as commercial products, e.g., Mar-gosom^®^ (active ingredient: oil of *Azadirachtin*), AjoNey^®^ (active ingredient: Essential oil of *Allium sativum* L.), Demize EC^®^ (active ingredient: *Citrus sinensis* L. oil limonene and linalool), Prev-Am^®^ (active ingredient: essential oil of *Thymus vulgaris*), Nico Dust^®^ (active ingredient: *Nictiana tabacum* L.) and By-O-reg + ^®^ (active ingredient: carvacrol/essential oil of *Origanum vulgare*) [28].

However, there is hope of overcoming the difficulties described here. Technological problems can be eliminated thanks to constantly improved methods that are based on nanotechnology [28,32,33]. Some companies have already developed nanoformulators, which are broad-spectrum pesticides, and are putting them on the market. As has been reported, many nanoformulations consisting of plant pesticides are biologically active and exert an influence on crop pests both before and after harvest [34,35,36,37,38].

The prospects for the usage of plant EOs and the chemical compounds that they contain for the production of packaging for food products are also promising. Nanoencapsulates, polymers and nanoemulsions are suitable for packaging. As a result, bioactive agents are slowly released, and the shelf life of the product is extended [39]. Efforts are also underway to unblock the placing on the market of products containing botanical pesticides, and the so-called “low-risk” ones have fewer stringent regulatory requirements [25,40].

The objective of the present study was to assess the efficacy of essential oil from common caraway and of L-carvone, which is a compound extracted from it, on the numbers of emigrating insects, mortality and emigration (repellence) for *S. oryzae*. It was assessed whether caraway essential oil and L-carvone affected *S. oryzae* as insecticides and/or repellents. After confirmation of effectiveness, the required concentrations and times of application were assessed. These studies augment the search that is currently being conducted in many countries with the aim of finding natural chemical components that show repellent effects towards this species of pest, without a concurrent negative effect on either human health or the environment.

The development of research on plant oils as plant protection products is particularly suitable for organic farming and for integrated pest management. Plant oils are biodegradable, have diverse physiological targets within insects and may thus delay the development of resistance among the insects [25].

## 2. Materials and Methods

The studies were conducted in laboratory conditions at 29° ± 1 °C with 60 ± 5% relative humidity (RH). Ten-day-old, adult beetles of *S. oryzae* used in the tests were obtained from breeding colonies kept under the same conditions as experimental colonies. The methodology of emigration (repellence) developed by Kłyś [41] was used in the emigration tests. Sets containing two plastic breeding containers were used: an inside container with a 28 cm^2^ of floor area, and an outside container with a 50 cm^2^ floor area. Forty grams of wheat grain were placed in each container. Wheat grain was the food and place for egg laying for *S. oryzae*. The containers were tightly sealed with perforated lids. The inside container had 30 holes of 1.5 mm in diameter separated by 1.5-cm spaces in the floor and sidewalls up to the level of the grain. Four 4-cm-high “screw inserts” were mounted onto the bottom of the inside container, allowing for the placement of the container above the wheat grain in the outside container, which prevented migrating beetles from returning to the inside container. The insects were placed in the inside container together with a circular ring of filter paper soaked with the caraway essential oil in the subsequent series of mass concentrations of 0.1, 0.5 and 1%, and with L-carvone in 0.05, 0.1, 0.5 and 1% concentrations. The insects were put into the inside container, numbering 40 pieces in each variant of the experiment and in each of its replications. The soaked filter paper disc had no contact with the wheat and was placed over the wheat. The circular ring of filter paper soaked in the substance remained effective throughout our study period. The emigration of insects from the internal to the external container was considered as the repellency. The caraway essential oil and L-carvone (liquid substances) were bought from Sigma–Aldrich and were applied without dilution. Their respective weight concentrations were calculated and weighed. The repellent effect, mortality and the numbers of emigrating insects were recorded after 1, 2, 3, 4, 5, 24 and 48 h of exposure. Each variant of the experiment was conducted in nine repetitions.

The estimates of the repellent effects were based on the emigration index, calculated as a percentage proportion of individuals emigrating when compared with the total number of individuals in the population. The calculations were made using the following formula:

$$\frac{{\bar{x}}_{el}+{\bar{x}}_{ed}}{{\bar{x}}_{l}+{\bar{x}}_{d}}\cdot 100\%$$

$\bar{x}$*_el_*—mean number of live migrants,

$\bar{x}$*_ed_*—mean number of dead migrants,

$\bar{x}$*_l_*—mean number of live individuals in both containers,

$\bar{x}$*_d_*—mean number of dead individuals in both containers.

The mortality index is the percentage proportion of dead individuals compared with the total number of individuals at a given time. It was calculated from the following formula [42]:

(1) $$\frac{{\bar{x}}_{d\;}}{{\bar{x}}_{d}+{\bar{x}}_{l}}\cdot 100\%$$

where:

$\bar{x}$*_d_*—mean number of dead insects,

$\bar{x}$*_l_*—mean number of live insects.

The control culture was carried out in the same temperature and humidity conditions, in the same set of containers and with the same number of replications as the experimental cultures. The substrate in the control culture was 40 g of pure wheat. The filter paper disc placed in the smaller vessel was not soaked with any substance. Forty beetles were released into the containers prepared in this way.

We have investigated whether there are statistically significant differences in the repellent effect of different concentrations of essential caraway oil and L-carvone on *S. oryzae*. The dependent variable is the insect emigration rate. Since the distribution of data in particular groups separated according to the concentration of the examined substances significantly differed from the normal distribution (Shapiro–Wilk test, *p* < 0.05), the analysis of variance (ANOVA) Kruskall–Wallis rank test was applied followed by the post hoc test, in this case a multiple comparison test [43]. The test probability level “*p*” and the significance level “α” were 0.05. The calculations were performed in the Statistica 13.3 program. 

## 3. Results

**Figure 1:**
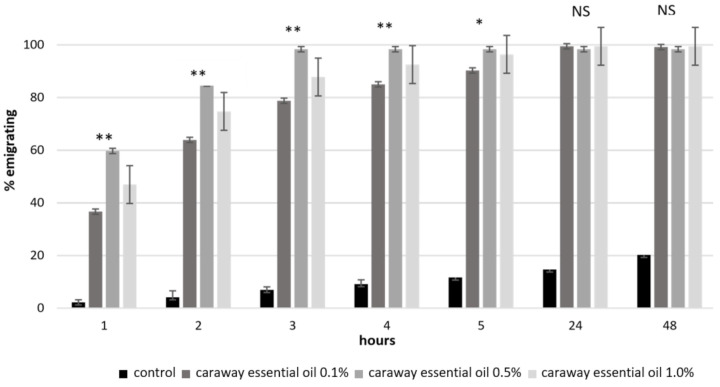
Repellency of *Sitophilus oryzae* caused by caraway essential oil (Statistically significant differences between the concentrations are marked with an asterisk. * 0.05 > *p* > 0.01; ** 0.01 > *p* > 0.001; NS—lack of significant differences; the figure indicates the mean of SE–standard error).

**Figure 2:**
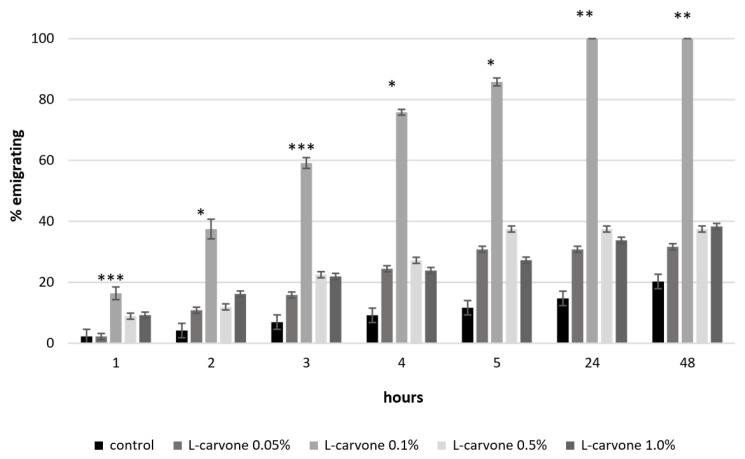
Repellency of *Sitophilus oryza* caused by L-carvone (Statistically significant differences between the concentrations are marked with an asterisk. * 0.05 > *p* > 0.01; ** 0.01 > *p* > 0.001; *** 0.001 > *p* > 0.0001; NS—lack of significant differences; the figure indicates the mean of SE).

The caraway essential oil applied in all the used concentrations (0.1, 0.5, 1%) resulted in a major repellent effect towards *S. oryzae*. After 1, 2, 3 and 4 h of study, the strongest repellence was confirmed by the highest values of the migration index, fluctuating from 60 to 98%, and was caused by the essential oil at a 0.5% concentration. In the control culture, the simultaneous emigration index fluctuated between 2 to 9%. After 24 and 48 h, the caraway essential oil in all the used concentrations resulted in very high emigration (repellence) among rice weevils. At that time, in the control culture, it amounted to only 13–19%. It was interesting to see that the highest repellence to *S. oryzae* in the initial four hours of studies was exerted for both the highest applied concentration and a lower one, i.e., 0.5% (Figure 1). A similar relationship was noted during the use of L-carvone: a 0.1% concentration resulted in the highest emigration of beetles (from 16 to 100%) in each of the analysed time intervals. The subsequent lower repellence (9–38%) was shown by L-carvone at a 0.5% concentration and then at a 1% concentration (7–22%) only after 1, 2, 3 and 4 h (Figure 2).

Analyzing the emigration results using the ANOVA Kruskall–Wallis test, statistically significant differences were found between the emigration of *S. oryzae* in the control culture and the emigration in the cultures with the addition of caraway essential oil at all concentrations and time intervals. Statistically significant differences in the emigration of *S. oryzae* under the influence of different concentrations of caraway oil and L-carvone in each time interval are marked with asterisks in Figure 1 and Figure 2.

The number of emigrants (the beetles repelled by the caraway essential oil used in the experiments at the three concentrations of 0.1, 0.5 and 1%) in each of the studied time intervals was markedly higher than that found in the control culture. After 1, 2, 3, 4 and 5 h, the highest number of beetles emigrating (respectively 24, 34, 39, 39 and 39) was noted after the effect of 0.5% caraway essential oil, and then after the application of 1% (respectively 21, 30, 35, 37 and 39) and 0.1% (respectively 15, 26, 31, 34 and 36). After 24 and 48 h, as many as 39 out of 40 beetles emigrated as an effect of the application of caraway oil at all the used concentrations. During the same time, only 5–7 beetles emigrated from the control culture.

**Figure 3:**
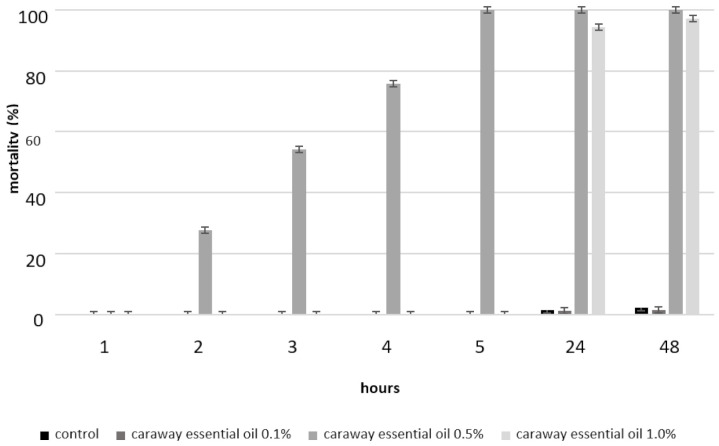
Mortality of *Sitophilus oryzae* caused by caraway essential oil (the figure indicates the mean of SE).

Of the three concentrations (0.1, 0.5 and 1%) used in the tests, the caraway essential oil caused the highest mortality among the *S. oryzae* individuals when applied at a 0.5% concentration. A statistical analysis of the *S. oryzae* mortality results showed statistically significant differences (*p* < 0.05) between the mortality in the control culture and the culture using caraway essential oil at a 0.5% concentration (from 2 to 24 h). At concentrations of 0.1 and 1%, the oil did not result in the mortality of the insects throughout the initial five hours of the experiments. It was only after 24 h that the mortality of the rice weevil population treated by caraway essential oil at 0.5 and 1% concentrations amounted to about 100%, and it was very low among the emigrants, i.e., the mortality indices fluctuated from 0.6 to 1.1% (Figure 3). The highest mortality among *S. oryzae* was obtained after applying the caraway essential oil at a concentration of 0.5%, and not after applying the highest concentration used in the tests.

**Figure 4:**
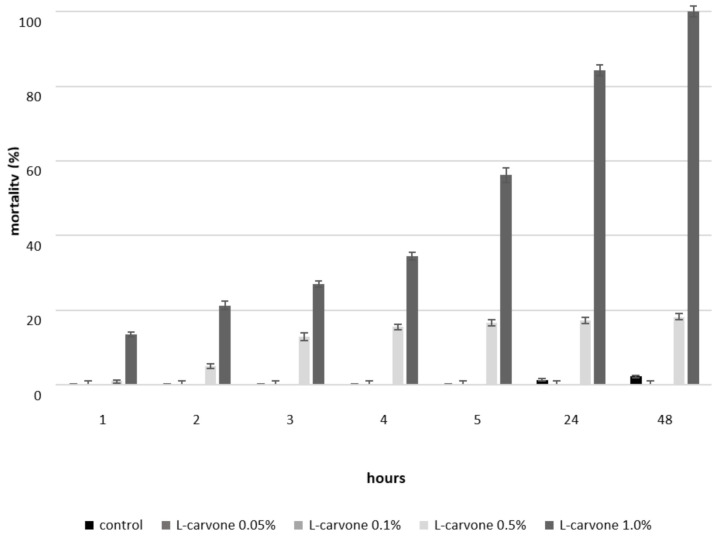
Mortality of *Sitophilus oryzae* caused by L-carvone (the figure indicates the mean of SE).

L-carvone at 0.05 and 0.1% concentrations did not cause mortality among *S. oryzae*. L-carvone at a 0.5% concentration resulted in a somewhat minor mortality of rice weevils from 0.9 to 18%. The highest mortality of *S. oryzae* was caused by L-carvone at a 1% concentration. After 1 h of exposure, it amounted to 10%, and after subsequent hours it rose to more than 50%. After 24 h, it amounted to more than 80%, and after 48 h there was a 100% mortality (Figure 4).

## 4. Discussion

A number of plant products and extracts were tested as repellents against *S. oryzae* with the use of various research techniques and with variable efficacy [19]. For example, the fastest repellent effect on rice weevil, after a mere 5 min, was exerted by the crude methanol extract of *Duabanga grandiflora* at a 0.252 mg/cm^2^ concentration, where 63% repellence was reached. After 4 h, the efficacy of repellence against the weevil was 100% [44].

In their studies, however, Tripathi and Upadhyay [45] obtained an efficacy of 91.1% repellence (PR) against adult *S. oryzae*, after 1 h when they applied leaf essential oil from *Hyptis suaveolens* at a concentration of 9.2 mg/cm^2^.

In another study, Nattudurai et al. [21] obtained 100% repellence against *S. oryzae* after 3 h, using a Y-tube glass olfactometer with essential oil isolated from leaves of *Toddalia asiatica* at a concentration of 20 µL. In their next experiment, Nattudurai et al. [46] observed that the same concentration of diethyl ether fruit extract also exhibited a 100% repellent activity against *S. oryzae*. An identical intensity of repellence (100%) against rice weevil was recorded by Ogendo et al. [47] after a 24 h exposure to oil and eugenol from *Ocimum gratissimum* at concentrations of 0.15% and 0.2% (*v/w*).

Using an olfactometer, Yoon et al. [48] analysed the repellent effects of six essential oils extracted from caraway, grapefruit, clary sage, strawberry, ylangylang and thyme white on *S. oryzae*. They found the highest repellent activities in (96.7%) carvone with limonene in caraway oil. Again, caraway oil displayed a high repellent efficacy (91.7%) against *S. oryzae* in a 10-µL dose after 24 h. In the study presented here, caraway essential oil at 0.1, 0.5 and 1% resulted after 24 h in a higher repellent efficacy (98–99%). However, L-carvon at a 0.1% concentration showed a 85% repellence against rice weevil as early as after 5 h, and it was 100% after 24 h. 

Elgizawy et al. [49] also evaluated the contact, fumigant toxicity and repellent activities of *Litsea cubeba* essential oil and two of its main active ingredients against adult *S. oryzae*. Essential oil, citral and D-limonene showed a strong contact toxicity against *S. oryzae* with LD50 7.51, 7.75 and 29.57 µg/L, while the fumigation toxicity against LD50 was 4.44, 4.89 and 16.68 µg/L, respectively. Essential oil, citral and D-limonene showed repellent properties at 81.83 and 53.30% at 2 and 4 h after the start of the study, respectively.

*Curcuma longa* L. leaf essential oil was also tested on adult *S. oryzae* for contact and fumigation toxicity and for its effect on offspring production. *S. oryzae* adults were very susceptible to fumigation activity, which amounted to LC50 11.36 mg/litre air. At a concentration of 40.5 mg/g food, the production of *S. oryzae’s* offspring was completely inhibited [50].

Kim et al. [51] assessed the insecticide activity and the inhibiting effect exerted on *Sitophilus oryzae* of acetylcholinesterase (AChE) from essential oils and compounds extracted from 10 species of plants of the family of Apiaceae. Among the plants included in the study, the essential oils obtained from *Anethum graveolens*, *Carum carvi* and *Cuminum cyminum* showed a strong fumigant toxicity against *S. oryzae*. The plants that were concerned also included *Carum carvi*. Among the compounds, (+)-carvone, (−)-carvone, cuminaldehyde, dihydrocarvone, linalool oxide, carveol, trans-anethole and neral also displayed higher toxicities against *S. oryzae* as fumigants. The strongest inhibitions towards acetylocholinoesterase were displayed by α-pinen, followed by β-pinen and limonene.

In our study, the essential oil from *Carum carvi* applied at a 0.5% concentration displayed an insecticidal action resulting in a 100% mortality among *S. oryzae* as early as after 5 h. L-carvone resulted in a 100% mortality of rice weevils at a 1% concentration after 48 h.

López et al. [52] also studied the effects of active substances contained in the essential oils obtained from *C. carvi* (carvon and limonene), *Coriandrum sativum* (linalool) and *Ocimum basilicum* (estragol) on the populations of *S. oryzae*, *R. dominica* and *Cryptolestes pusillus*. The most effective monoterpenoid against *S. oryzae* was carvon (1364 ppm) in combination with camphor (131 ppm), where 100% of the beetles were dead after 24 h. Other mixtures of active substances, whose main component was caraway oil, caused a high mortality in both rice weevils and the remaining two species (approx. 90–100%). Against *S. oryzae*, the mortality index in the application of linalool (1723 ppm) combined with camphor (185 ppm) reached 63%. Against *R. dominica* and *C. pusillus,* the mortality indexes were 96% and 100%, respectively. Estragol affected the activities of rice weevil in a variable and ambiguous way. López et al. [52] emphasised that the principal active substances contained in the plants that were used, applied both separately or together with other substances, worked both as insecticides and as repellents against the tested storage pests.

The most interesting results obtained in our research include those that indicate the highest repellent effects of caraway essential oil and L-carvon on *S. oryzae* at both the highest and lower applied concentrations, i.e., 0.5% and 0.1%, respectively. The explanation for the mechanism of this phenomenon in insects requires further physiological and biochemical studies. Similar results were obtained with respect to other arthropod species, e.g., mosquitoes, biting flies, fleas and ticks, when applying repellents based on DEET and permethrin. DEET is a repellent with a wide spectrum of action against arthropod bites. Although the protection against arthropod bites provided by DEET is proportional to the logarithm of the dose, the higher concentrations of DEET ensure that the protection lasts longer; however, in the range up to 50%, concentrations above 50% do not increase the efficacy of DEET [53]. Furthermore, tests carried out on ticks showed that permethrin did not reduce the reproductive rate of females at the highest applied dose (12.5 μg) but at a lower dose of 6.25 μg [54]. Additionally, this relationship has not yet been elucidated in ticks.

As Weaver and Subramanyam [55] say, the used bioassay approaches should address all potential modes of action (contact, oral, fumigant, repellent effects) on target insect pests.

In our research on the effect of *C. carvi* on *S. oryzae*, we also checked the effect of contact and the oral effects, but their effectiveness was not as high as in the case of essential oil and L-carvon.

This article showed a strong repellent effect of both caraway essential oil and the compound present in a large amount (approx. 60%)—L-carvone—on *S. oryzae*. *C. carvi* EO was also found to be highly insecticidal. At the present stage of research, it can be said that both of these compounds are good candidates for practical applications in preventing the occurrence of this pest in storage areas, as well as in eliminating *S. oryzae* by scaring cereals and food products from stored grain. Deterring *S. oryzae* from products will reduce not only quantitative but also qualitative losses caused by contamination of the stored cereal grain, for example through dead individuals or excrement. The fact that *C. carvi* is a spice plant and therefore not toxic to humans is also not insignificant. Cumin EO and L-carvone could be used as repellants and insecticides as one element in an integrated pest management program against *S. oryzae*.

These compounds could also be used together with other botanical pesticides to control this pest. In the future, we should also think about the use of caraway EO and L-carvone in nanotechnological research in terms of their practical applications, e.g., for the production of packaging for food products and cereal grains, which will protect them against the attack of *S. oryzae*. It is hoped that all these measures would help limit the damage caused by rice weevil.

## 5. Conclusions

In conclusion, the greatest repellent effects on *S. oryzae* were caused by lower doses of caraway essential oil and L-carvone, which is a new discovery related to plant compounds used against stored pests. This differs from the results that were so far obtained in relation to stored pests, in which repellency increased with increasing concentrations. Similar results to our research were obtained with respect to other arthropod species, e.g., mosquitoes, biting flies, fleas and ticks, when applying repellents based on DEET and permethrin.
